# Role of chitosan in titanium coatings. trends and new generations of coatings

**DOI:** 10.3389/fbioe.2022.907589

**Published:** 2022-07-22

**Authors:** Nansi López-Valverde, Javier Aragoneses, Antonio López-Valverde, Cinthia Rodríguez, Bruno Macedo de Sousa, Juan Manuel Aragoneses

**Affiliations:** ^1^ Department of Medicine and Medical Specialties, Faculty of Health Sciences, Universidad Alcalá de Henares, Madrid, Spain; ^2^ Department of Surgery, University of Salamanca, Instituto de Investigación Biomédica de Salamanca (IBSAL), Salamanca, Spain; ^3^ Department of Dentistry, Universidad Federico Henríquez y Carvajal, Santo Domingo, Dominican Republic; ^4^ Institute for Occlusion and Orofacial Pain, Faculty of Medicine, University of Coimbra, Polo I‐Edifício Central Rua Larga, Coimbra, Portugal; ^5^ Faculty of Dentistry, Universidad Alfonso X El Sabio, Madrid, Spain

**Keywords:** titanium dental implants1, osteointegration2, bioactive surfaces3, chitosan coating4, future direction5

## Abstract

Survival studies of dental implants currently reach high figures. However, considering that the recipients are middle-aged individuals with associated pathologies, research is focused on achieving bioactive surfaces that ensure osseointegration. Chitosan is a biocompatible, degradable polysaccharide with antimicrobial and anti-inflammatory properties, capable of inducing increased growth and fixation of osteoblasts around chitosan-coated titanium. Certain chemical modifications to its structure have been shown to enhance its antibacterial activity and osteoinductive properties and it is generally believed that chitosan-coated dental implants may have enhanced osseointegration capabilities and are likely to become a commercial option in the future. Our review provided an overview of the current concepts and theories of osseointegration and current titanium dental implant surfaces and coatings, with a special focus on the *in vivo* investigation of chitosan-coated implants and a current perspective on the future of titanium dental implant coatings.

## Introduction

In the last 50 years, dental implants have become a predictable treatment option for the replacement of missing teeth, improving the quality of life and masticatory function of patients rehabilitated using them (Jofre et al., 2013; Hartlev et al., 2014; Tarnow, 2014; Buser et al., 2017).

There are currently about 1,500 different implant systems in terms of topography, wettability, chemistry, and surface modification (Le Guéhennec et al., 2007; Junker et al., 20092009). These characteristics contribute to the biological processes occurring during osseointegration by direct interaction with host osteoblasts in bone formation (Le Guéhennec et al., 2007; Dohan Ehrenfest et al., 2010; Pattanaik et al., 2012; Ogle, 2015; Hotchkiss et al., 2016; Wennerberg et al., 2018).

In general, long-term studies report excellent results in terms of the survival rate of dental implants. After 20 years in operation, the overall cumulative survival rate ranges from 89.5 to 99%, according to different studies (Horikawa et al., 2017; Chrcanovic et al., 2018). However, considering that most patients who undergo dental implant treatment are of a certain age (50–60 years) (Guillaume, 2016) in which coexisting pathologies (diabetes mellitus, osteoporosis, bisphosphonate treatments) can affect bone quality and quantity, making it necessary to modify the bioactive surface to accelerate and ensure osseointegration after implant insertion (Gil et al., 2021). On the other hand, surface modification becomes necessary to accelerate osseointegration and to achieve more comfortable and faster prosthetic loading protocols. For all these reasons, biomedical research on surface modifications of dental implants is focused on achieving bioactive surfaces that guarantee long-term bone-implant contact (Bosshardt et al., 2017).

Today’s rigorous manufacturing processes in modern dental implantology have brought about a true technological revolution. The breakthrough in the treatment of edentulism, although it has not been given the deserved relevance as other novel surgical procedures, greatly influences the physiological and psychological state of patients and improves their quality of life (Guillaume, 2016; Fonteyne et al., 2021).

Chitosan (Cht) is an FDA-approved copolymer that has demonstrated properties such as bioactivity, biocompatibility, biodegradability, non-toxicity and broad-spectrum antimicrobial activity against both gram-positive and gram-negative bacteria. On the other hand, it is important to highlight the prominent role of chitosan-based scaffolds in combination with natural biomolecules and drugs in bone regeneration (Fakhri et al., 2020).

Our review provided an overview of the current concepts and theories of osseointegration and current titanium dental implant surfaces and coatings, with a special focus on the *in vivo* investigation of chitosan coatings and a future perspective on coatings.

## Titanium

The history of titanium (Ti) as a biomedical material began in the 1940s. Bothe et al. implanted titanium together with other metallic materials in laboratory animals, re-porting its good tolerance due to its excellent resistance to corrosion in biological fluids; Beder et al. proposed the use of Ti for intraoral implants, using a canine model (Clarke and Hickman, 1953; Beder and Ploger, 1959; Nakajima and Okabe, 1996).

Currently for the manufacture of orthopedic and dental implants, four grades of pure Ti are used depending on their oxygen and iron content. Apart from pure Ti, Titanium-Aluminum-Vanadium alloy (Ti-6Al-4V, Ti6-4) and Ti-grade 5 are the most commonly used for biomedical applications. Ti6Al4V ELI (Grade 23) is an alloy of titanium with aluminum and vanadium. It is a purer version of Ti6Al4V (Grade 5). The content of interstitial elements (iron, oxygen and carbon) in this alloy are strictly controlled and limited during the melting process. This purity gives it superior mechanical properties and increased fatigue strength. It has excellent biocompatibility with the human body and is one of the most widely used dental implants to restore function (Jujur et al., 2020).

## The concept of osseointegration

The pioneer of modern implant dentistry, Professor Brånemark of the University of Gothenburg (Sweden), performed the first preclinical studies in the 1960s, describing the phenomenon of osseointegration. The ‘Brånemark team’ (Tomas Albrektsson, Ragnar Adell, Ulf Lekholm and Torsten Jemt) was the first to propose the concept of osseointegration of a metallic biomaterial implanted in bone, demonstrating that biocompatibility and bone-Ti bonding were the main biological properties of this metal. This aspect led them to define the concept of osseointegration of titanium as ‘the direct structural and functional connection between the ordered living bone and the surface of a load-bearing implant’ (Brånemark et al., 1977; Adell et al., 1981; Albrektsson et al., 1981). However, the biological bone-Ti interaction can be found in the scientific literature under different terminologies such as ‘bone ankylosis’, ‘bone union’, ‘osteotolerance’ etc. that define more precisely the bone-implant relationship (Lang, 2019).

Cornell & Lane defined osteoconduction a three-dimensional process of ingrowth of capillaries sprouting from a bone bed, perivascular tissue and osteoprogenitor cells into the three-dimensional structure of a porous implant, which is used as a guide to cover a defect with bone tissue (Cornell and Lane, 1998). Osteoinduction is defined by Barradas et al. as the induction of undifferentiated mesenchymal stem cells, which are not yet committed to the osteogenic lineage, to form osteoprogenitor cells and produce bone in heterotopic sites (Barradas et al., 2011; Weber, 2019). However, the actual mechanism of the osseointegration process remains unknown (López-Valverde et al., 2020) although, in recent years, different novel theories have been proposed to explain it:

### Osteosufficiency/osteoseparation theory

Kota and Zarb (Koka and Zarb, 2012) have proposed a rational theory in which host biology and implant characteristics are considered two distinct entities that are expected to interact and coexist for decades and caution that although short-term studies, animal models and *in vitro* work may provide clues, long-term human observation is the definitive reason. Some factors predisposing to a state of osteoinsufficiency create little controversy, e.g., uncontrolled diabetic; others, such as parafunctional habits, occlusal overload, certain drugs, or bacterial aggression are controversial.

### Brain-bone axis theory

The central nervous system, and more specifically the hypothalamus, has an important regulatory role in the functions of peripheral tissues and in the processes of bone healing and remodeling (Ruan et al., 2014; Kim et al., 2015). Recent studies have also demonstrated important links between the central nervous system and the immune system, which in turn plays a key role in peri-implant bone healing (Elefteriou et al., 2014). Other studies have suggested that impaired osseointegration and dental implant failures may be associated with the use of antidepressant drugs (Abu Nada et al., 2018). Some researchers have even gone so far as to propose the gut-brain-bone axis, in which the gut would drive bone physiology through the regulation of key hormones that are originally synthesized in the brain. And in this aspect, the oral microbiota would play an important role (Quach and Britton, 2017).

### Foreign body reaction theory

Any type of implant is considered a foreign body, to which the organism reacts by activating the immune and inflammatory systems, whereby the defense cells (neutrophils, lymphocytes, proinflammatory reactive macrophages and osteoclasts) react by engulfing the foreign body. In this situation, reparative cells (fibroblasts and osteoblasts) are activated and help repair and remodel tissues and protect them from further destruction, however, when a foreign body is too bulky to be engulfed by immune cells, a fibrous or osseous encapsulation process develops around it (Carcuac et al., 2013; Miron et al., 2016; Naveau et al., 2019). Trindade et al. and Albrektsson et al. defined osseointegration as a foreign body reaction, an immunomodulated, multifactorial and complex healing process involving various cells and mediators, hypothesizing that the primary etiology of crestal bone loss around osseointegrated implants would be a change in the inflammatory equilibrium (foreign body equilibrium). Such an inflammatory response would be caused by sudden changes in the loading situation, or by alterations of the foreign body itself, in the form of accidental tissue dispersion, and that bacterial colonization, now classically considered as a triggering factor for peri-implant bone loss, could be secondary to such alterations (Trindade et al., 2016; Trindade et al., 2018).

## Titanium surface modifications

**FIGURE 1:**
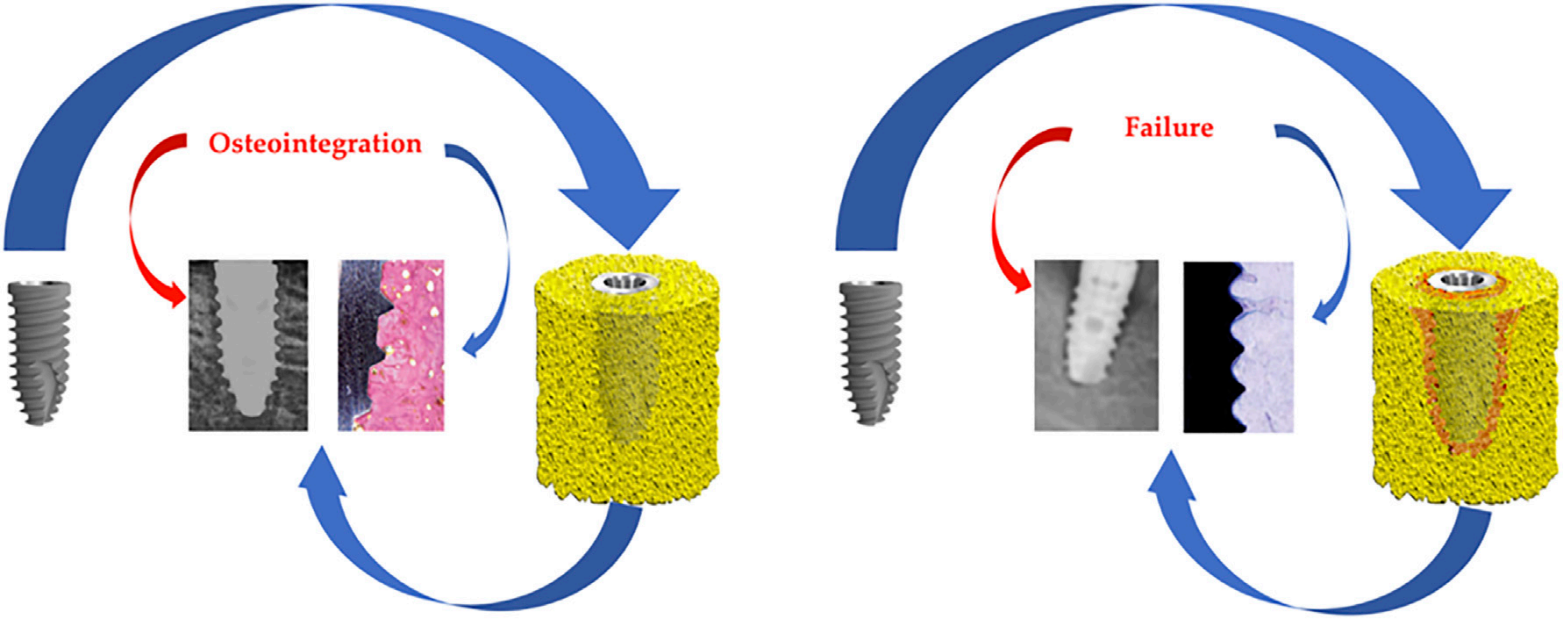
Osseointegration and failure.

After implantation, titanium comes into contact with biological fluids and tissues, and two types of response may occur: either the formation of a fibrous soft tissue capsule around the implant that does not guarantee adequate biomechanical fixation and leads to clinical failure, or direct bone-to-implant contact without an intermediate connective tissue layer, which is known as osseointegration (Brånemark et al., 1977) (Figure 1).

Most dental implants are manufactured with grade 4 Ti, as it is stronger than other grades, however, titanium alloys are mainly composed of Ti6Al4V (Titanium grade 5-Aluminum-Vanadium), with higher elastic modulus and better fatigue properties than pure titanium (Glied and Mundiya, 2021).

**FIGURE 2:**
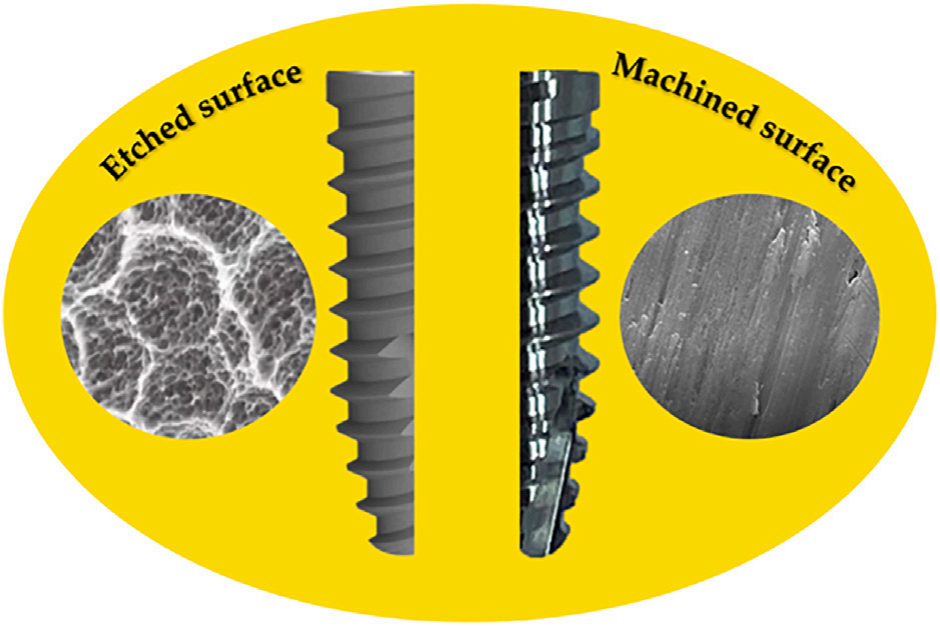
Engraved and machined surface of Ti implants.

The implant surface is now considered to play a key role in clinical success. The surface roughness of Ti implants affects the osseointegration rate and biomechanical fixation (Ferraris et al., 2011). Ti implants with SLA (Sandblasted, Large-grit, Acid-etched) etched surfaces show superior bone-to-implant contact (50–60%) compared to other surface modifications, and the suitability of this type of etching in terms of overall osteogenic performance has been demonstrated *in vivo* (Ogle, 2015; Ren et al., 2021) (Figure 2).

The degree of surface wettability (range 0° hydrophilic to 140° hydrophobic) is a matter of controversy among different researchers; Buser et al. (Buser et al., 2004) proposed that hydrophilic SLA surfaces generate greater bone-to-implant contact than normal SLA. However, earlier *in vivo* studies by Carlsson et al. and Wennerberg et al. found no difference (Carlsson et al., 1989; Wennerberg et al., 1991). Nevertheless, new findings on wetting and nanoroughness are driving current research in this exciting field (Rupp et al., 2018).

Currently, surface roughening is the most commonly used technique in practice, however, titanium implants with microscale and/or nanoscale surface topographical features have moved from novelty to commodity and are advancing in the field of the implant industry. Certain researchers have reported that the failure resistance of dental implants is influenced by the acid-etched surface; furthermore, it is known that Ti etched, SLA-type surfaces have a mean value, after 6 weeks, of 50–60% bone-to-implant contact, compared to the Ti plasma sprayed titanium surface, which had only a mean value of 30–40% (Ong and Chan, 2000; Socorro-Perdomo et al., 2022).

To ensure a high quality of coatings, the importance of surface pretreatment prior to the deposition work has to be taken into account. Despite the high number of studies performed to date on the plasma spraying method, the literature results demonstrate the difficulties in deciding the optimal value of surface roughness to improve osseointegration and decrease bacterial adhesion (Jemat et al., 2015).

Implant surface modifications by methods based on the immobilization of biologically active organic molecules on Ti and Ti6Al4V surfaces have attracted much interest recently (Panayotov et al., 2015).

Human defensins are normally produced by neutrophil granulocytes and epithelial cells. The alpha defensins, were originally identified as small antibiotic peptides, termed human neutrophil peptides (HNP-1, HNP-2, HNP-3) (Jarczak et al., 2013). Notably, HNP-1 has been detected in saliva samples from patients with oral cancer and patients with other oral pathologies. Human beta-defensins are synthesized by epithelial cells lining the oral mucosal surfaces. Beta-defensin is secreted in saliva and it has been found that certain inflammatory stimuli could significantly increase beta-defensin expression in gingival keratinocyte cultures (Cunliffe, 2003). Pfeufer et al. (Pfeufer et al., 2011) observed that coating Ti surfaces with human beta-defensin was effective against Escherichia coli, a gram-negative bacterium associated with implant failure (Alshammari et al., 2021).

Histatins are antimicrobial peptides secreted in human parotid saliva, which have been attributed an important role in wound healing *in vitro* (Pal et al., 2014). Certain studies have highlighted the role of these peptides in the osseointegration of Ti dental implants. Van Dijk et al. immobilized histatin peptide on the surface of Ti and found that it enhanced osteoblast cell adhesion (Van Dijk et al., 2017). Makihira et al. tested the performance of Ti implants coated with a histatin-derived peptide on edentulous ridges of dogs; histological analysis and microcomputed tomography showed increased trabecular bone formation around the coated implants versus untreated implants (Makihira et al., 2011). Siwakul et al. (Siwakul et al., 2021) evaluated cell adhesion, proliferation, osteogenesis-related genes and alkaline phosphatase activity on histatin-coated Ti surfaces; the results showed that the adhesion of cells to the histatin-coated group achieved cell proliferation significantly histatin achieved significantly higher cell proliferation.

Sugawara et al. (Sugawara et al., 2016) described a method that allowed adhesion of human gingival epithelial cells to a smooth Ti surface by a protease receptor 4 activating peptide, based on the idea that peri-implantitis could result from a lack of epithelial seal at the peri-implant collar; the coating produced a rapid aggregation of platelets on the Ti surface. Other studies have demonstrated the induced epithelial barrier function to prevent bacterial adhesion, penetration, and invasion on Ti (Maeno et al., 2017). Local production of antimicrobial peptides may contribute to the barrier function of the epithelial seal formed around the transmucosal part of dental implants; in this regard it is noteworthy that certain bacteria such as *Fusobacterium nucleatum*, an anaerobic member of the oral pathogens associated with periodontitis, induced the production of beta-defensin-2 in human gingival epithelial cells (Krisanaprakornkit et al., 2000). This type of research on the immobilization of peptides on Ti surfaces could prevent bacterial colonization of implants and facilitate the initial phase of osseointegration (Table 1).

**TABLE 1:** Innovative surfaces based on organic components.

Surface coating	Sustrate	Outcome	Study
Human beta-defensin	Ti	Effective against Escherichia coli	Pfeufer et al. (Pfeufer et al., 2011)
Histatins	Ti	Enhance osteoblast cell adhesion	Van Dijk et al. (Van Dijk et al., 2017)
	Ti	Increased trabecular bone formation around coated implants	Makihira et al. (Makihira et al., 2011)
	Ti	Promote cellular activities around dental implants	Siwakul et al. (Siwakul et al., 2021)
Peptides	Ti	Adhesion of human gingival epithelial cells to a smooth titanium surface. Epithelial sealing at the implant neck. Platelet aggregation on the titanium surface	Sugawara et al. (Sugawara et al., 2016)
	Ti	Induced epithelial barrier to prevent bacterial adhesion, penetration and invasion on titanium. Prevent adhesion, penetration and invasion of Escherichia coli bacteria	Maeno et al. (Maeno et al., 2017)
	Ti	Certain oral pathogens associated with periodontitis induce the production of beta-defensin-2 in human gingival epithelial cells	Krisanaprakornkit et al. (Krisanaprakornkit et al., 2000)

## Chitin and chitosan

Chitin is a carbohydrate found mainly in the exoskeleton of crustaceans and some fungi. Albert Hofmann first described its structure, although as early as 1859, Rouget treated chitin with a hot potassium hydroxide solution, leading to the discovery of chitosan (Cht) and laying the foundations for its production (Crini, 2019; Mohan et al., 2022). The deacetylation process increases the interaction between Cht and cells by increasing the number of positive charges, thus improving its biocompatibility (Hamed et al., 2016). It does not produce antigenic response and possesses anti-inflammatory properties. Its hemostatic power is an important feature, as it can induce platelet adhesion and aggregation and activate blood coagulation. Thus, Cht can control bleeding by adsorbing plasma and coagulating red blood cells (Keast DH Janmohammad, 2021; Ferraris S et al., 2022).

### Chitosan titanium dental implants coatings

Surface treatments and coatings of dental implants are of great importance in the osseointegration process. Cht has excellent ability to bind to metal, which could improve the mechanical strength and increase the durability of titanium implants. However, solubility in aqueous solutions depends on pH. At neutral pH, most chitosan molecules will lose their charge and precipit from solution. Certain researchers have highlighted the adhesion strength of Cht coatings to Ti surfaces as unsuitable for clinical applications. Ferraris et al. (Ferraris S et al., 2022) has recently proposed a chemical pretreatment of the Ti substrate, by three methods: in the first one (“direct coating”) he immerses the Ti6Al4V samples in the solution; in the second one, he activates the surface by tresyl chloride and in the third one he performs a treatment with polidopamine, concluding that the best result is obtained with the direct coating at acid pH on a pretreated Ti substrate, due to the nanometric porosity of the substrate and to the strong electrostatic attraction between the chitosan and the hydroxyl groups. In this way, mechanically and chemically stable coatings are obtained.

Natural or biopolymer-based composites containing chitin or chitosan have advantages such as biocompatibility and biodegradability, which are essential for bone tissue engineering. Carboxymethylation, quaternization, sulfonation and phosphorylation are the most common methods to overcome the insolubility of Cht and to enhance its antimicrobial effect (Qin and Li, 2020).

**FIGURE 3:**
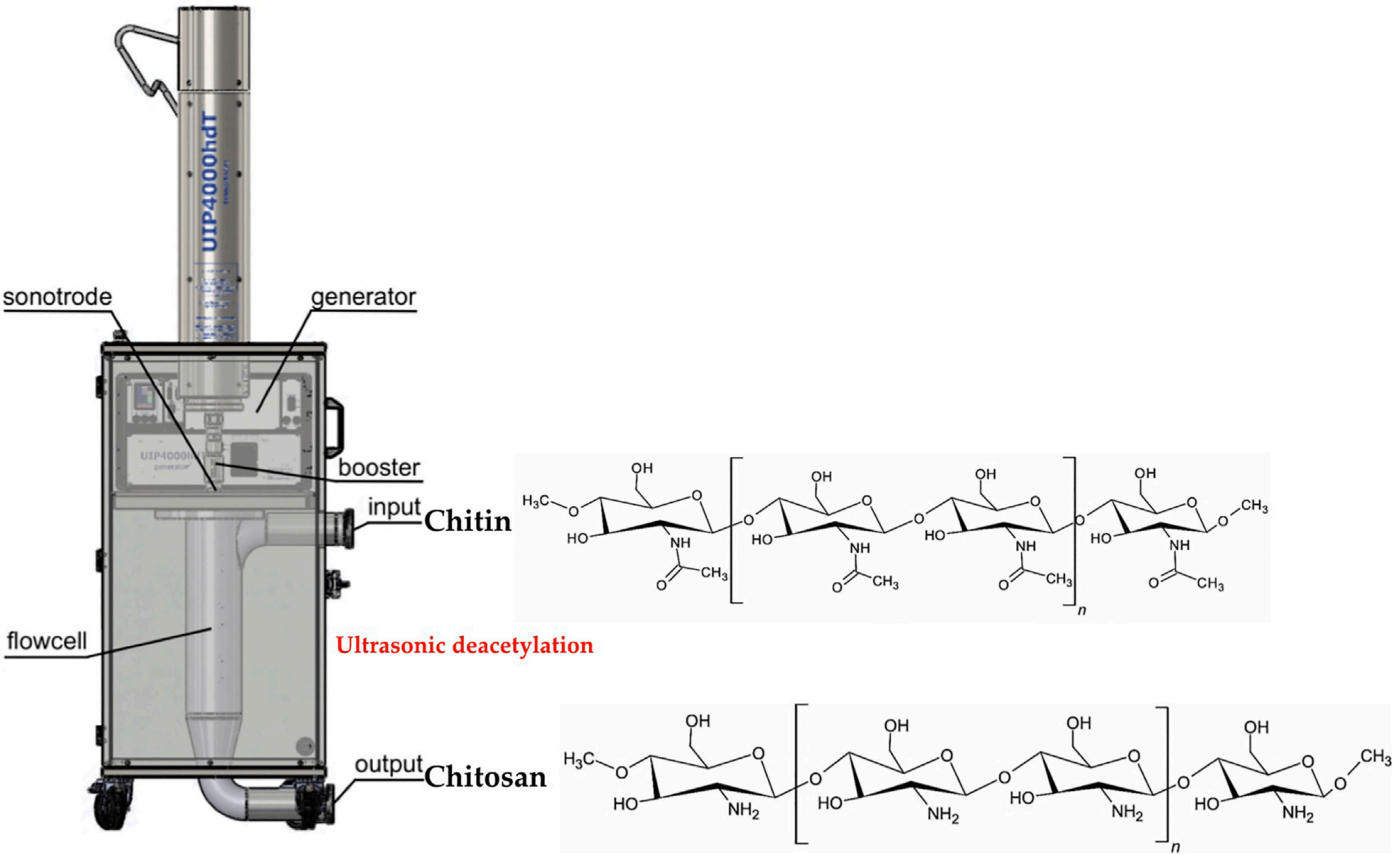
Chitin deacetylation process.

Cht is produced by the N-deacetylation of chitin. The application of high-power ultrasound significantly intensifies the deacetylation process (removal of an acetyl group) of chitin, resulting in low molecular weight, high quality Cht by rapid treatment at low temperature (Figure 3).

It has been shown that, deacetylated Cht chemically binds to titanium surfaces, although the bond strength is lower than that of calcium phosphate coating; however, enhanced osteoblast growth and attachment has been reported on Cht-coated titanium (Bumgardner et al., 2003a). Despite this, *in vitro* and *in vivo* studies disagree as *in vitro* studies show that osteoblasts do not adhere to chitosan in normal cell cultures, requiring some pretreatments such as serum soaking or fibronectin coating (Saccani et al., 2019).

### Preparation of the different types of chitosan coating

The characteristic features of Cht, such as being cationic, hemostatic and insoluble at high pH, can be completely reversed by a sulfation process that can make the molecule anionic and water soluble, and also add anticoagulant properties (Suh and Matthew, 2000). On the other hand, due to its high molecular weight and linear structure, it has excellent viscosity. The higher viscosity of the Cht solution depends on the concentration, the decrease in temperature and the increase in the degree of deacetylation; this influences the elongation at fracture and tensile strength, all of which is of great importance in Cht coatings of dental implants by immersion, which would be more resistant to the forces provoked during the insertion phase in the bone tissue bed (Barton et al., 2014); however, studies investigating the influence of the degree of deacetylation on the properties of chitosan are contradictory. Foster et al. observed that variation in the degree of deacetylation could be responsible for the contrasting trends in chitosan properties. (Foster et al., 2015). At the same time, viscosity would enhance biological and healing properties, as well as biodegradation and osteogenic capacity (Gérentes et al., 2002). Other modifications of Cht, with the aim of reducing the drawbacks it presents (mechanical properties and antibacterial activity for biomedical applications), have involved chemical modification. Modifications of Cht, such as the incorporation of carboxymethyl, imizadolyl or methyl pyrrolidone, have been shown to improve osteoinductive properties and result in increased antibacterial activity (Alves and Mano, 2008; Jayakumar et al., 2010). Other strategies to compensate for its limitations have consisted of combining it with other natural polymers such as alginate, silk, or chitin (Mohire and Yadav, 2010; Rahmani et al., 2018; Quesada et al., 2020; Moenne and González, 2021).

**FIGURE 4:**
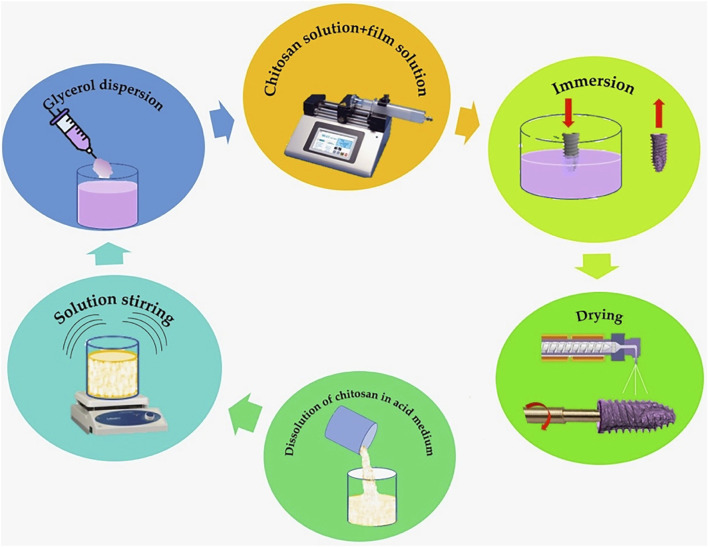
Modified Vakili and Zhang coating scheme for the immersion procedure.

Cht as a titanium coating has been studied in depth by different researchers (Bumgardner et al., 2003a; Abarrategi et al., 2008), however, few *in vivo* studies have been published (Bumgardner et al., 2007; Kung et al., 2011; Takanche et al., 2018; Zhang et al., 2020; López-Valverde et al., 2021a; López-Valverde et al., 2022). On the other hand, when preparing Cht coatings for silanization and attachment to the Ti substrate, toxic reagents such as 3 isocyanatopropyltriethoxysilane and glutaraldehyde are used; moreover, these techniques involve complex processing that hinders the deposition of the coating and limits its clinical applicability. Grosso described an immersion coating method for preparing films on substrates from liquid solutions. This method facilitates the possibility of fine-tuning the amount of material that can be deposited, and thus the thickness of the final film; moreover, dip coating is an inexpensive way to deposit thin layers from chemical solutions with relatively fair control of the layer thickness (Grosso, 2011). The kinetics of dip coating is based on a continuous flow condition and the coating thickness is determined by the competition between viscous force, surface tension, gravity and substrate withdrawal rate (Nguyen du et al., 2014). Zhang et al. (Zhang et al., 2020) demonstrated that porous Ti with a Cht/hydroxyapatite coating could promote osteoblast-like cell proliferation and differentiation and osseointegration in a rabbit femur model. For the coating process, they resorted to a dilution of Cht/hydroxyapatite composite by dissolving 1.98 g of Ca(NO3)2–4H2O and 0.66 g of KH2PO4 in distilled water, along with 1 g of Cht powder in 100 ml of 2% acetic acid. The 2 solutions were mixed to prepare a composite Cht/hydroxyapatite coating on the porous titanium surface using an electrochemical deposition method described by Pang et al. (Pang and Zhitomirsky, 2007). Takanche et al. (Takanche et al., 2018) used a rat mandible osteoporotic model, where they implanted Ti devices coated with Cht-gold nanoparticles, obtaining an increase in osteogenesis and inhibition of osteoclastogenesis. Bumgardner et al. (Bumgardner et al., 2007) on a rabbit tibia model, implanted Ti devices coated with deacetylated Cht (1wt% deacetylated chitosan 92.3% in 1% acetic acid) melted in solution and adhered to rough Ti pins by their own method (Bumgardner et al., 2003a; Bumgardner et al., 2003b), using calcium phosphate-coated Ti pins and uncoated Ti pins as controls. The coating with Cht was performed by solution casting and silane reactions under acidic conditions. They used a 1% Cht solution in 1% acetic acid, poured over the samples and allowed to air dry for 7 days. The resulting layers, 10–15 µm thick, were neutralized with a weak base and rinsed with plenty of deionized water. Histological evaluations of the tissues in contact with the Cht-coated pins indicated a minimal inflammatory response and a healing sequence with bone tissue formation, followed by the development of laminar bone. Kung et al. (Kung et al., 2011) resorted to subcutaneous implants in a rat model, using two types of Cht with a degree of deacetylation >90%. The solution used consisted of 15 mg of Cht powder in 10 ml of vitamin C solution to obtain a 0.15% Cht solution with which they soaked type I collagen membranes that they then wrapped around Ti mini-implants, obtaining ectopic new bone formation in the area of the Cht-coated implants. The method, used by us in previous studies (Pang and Zhitomirsky, 2007; Nguyen du et al., 2014), resorted to a film-forming solution of Cht dissolved in an acid medium, on a dog jaw model; they compared implants with surfaces coated with Cht, by means of a procedure of immersion in acid solution and film-forming solution, with uncoated implants, type SLA. In our opinion, this is a suitable procedure for the coating of dental implants with Cht. The procedure, described by Vakili et al. (Vakili and Asefnejad, 2020), complemented with the procedure described by Zhang et al. (Zhang et al., 2019) for the film-forming solution, is performed by dissolving 0.5% (w/v) Cht in a 0.5% (v/v) acid solution and stirring the solution for 12 h on a magnetic stirrer. The film-forming solution is prepared following the procedure described by Zhang et al.; glycerol (0.4 g) is dispersed in 80 ml acetic acid (1%, w/v) by stirring for at least 12 h (4°C). The prepared Cht solution is added to the film-forming solution using a syringe pump (Infusomat^®^ Space, Braun, Barcelona, Spain), at a rate of 50 ml/h, stirring by means of a mechanical stirrer at 800 rpm. Functionalization of the Cht-coated implants was performed by immersion in the prepared solution. The functionalized implants were then dried at 25°C with a relative humidity of 50% for the formation of a uniform film. (Figure 4).

Four of the *in vivo* studies reviewed (Bumgardner et al., 2007; Kung et al., 2011; Takanche et al., 2018; Zhang et al., 2020; López-Valverde et al., 2021a; López-Valverde et al., 2022) used coating formulations, in our opinion, of a certain complexity in terms of clinical applicability. The fifth study and sixth studies (López-Valverde et al., 2021a; López-Valverde et al., 2022) used a coating process described by Vakili et al. (Vakili and Asefnejad, 2020) and Zhang et al. (Zhang et al., 2019), with slight modifications, of easy applicability (Table 2). Grosso (Grosso, 2011) recommends immersion methods as a simple and inexpensive coating strategy, easily applicable in preclinical studies applicable in humans. The CAMARADES Research Group (University of Edinburgh) reported that simple strategies for preclinical studies in general, save more than 30% of in-research expenditure ($5.8 billion) (CAMARADES, 2022).

**TABLE 2:** Characteristics of *in vivo* studies.

*In vivo* studies	Aplicability	Cost	**Difficulty of coating preparation**	Substrate	Coating and method	Outcomes
Zhang et al. (Zhang et al., 2020)	+ +	+ + +	+ + +	Porous Ti	Cht/HA composite coating	Osteoblast-like cell proliferation and differentiation
					Electrochemical deposition	
Takanche et al. (Takanche et al., 2018)	+	+ + +	+ + +	Ti	Chitosan-gold nanoparticles	Increased osteogenesis and inhibition of osteoclastogenesis
					Graft-on technique	
Bumgardner et al. (Bumgardner et al., 2007)	+ +	+ +	+ +	Ti	1 wt% of 92.3% deacetylated chitosan in 1% acetic acid	Minimal inflammatory response and development of lamellar bone
					Solution casting	
Kung et al. (Kung et al., 2011)	+	+ +	+ +	Ti	Two types of chitosan with molecular weights of 450 and 750 kDa and with deacetylation degree	New bone formed ectopically
					> 90%	
					Enveloped membranes	
López-Valverde et al. (López-Valverde et al., (2021a); López-Valverde et al., (2022))	+ + +	+	+	Porous	Pure chitosan acid solution	Higher bone density compared to the conventional etched surface group
				Ti6Al4V	Immersion	

+ low; + + moderate; + + + high; HA, hydroxyapatite.

In any case, we have not found defined in the literature the optimal amount of Cht for coating Ti dental implants, although some research has hypothesized that it may not be important for the Cht coating to persist in the long term once a good bone-implant interface has been established. It also seems to be unclear whether it would be necessary for the chitosan polymeric material to possess high bond strength (Bumgardner et al., 2007).

Cht coatings on Ti-6Al-4V substrates *in vitro*, which showed improved mechanics, better cell adhesion and viability of human osteoblastic cells, were obtained by homogeneous coatings of 770–800 nanomicrons es-thickness, crack-free and well adhered (Gopi et al., 2014; Moskalewicz et al., 2015).

The layer-by-layer technique has many advantages over other multilayer fabrication methods, as no complicated instruments are needed for the process, which makes it affordable in practice; moreover, deposition by layer-by-layer is independent of the shape and size of the substrate. Song et al. (Song et al., 2020) used the layer-by-layer approach to modify the titanium substrate, obtaining smooth multilayer coatings that promoted osteogenic differentiation of MG63 osteoblast-like cells.

Casting is another simple method of depositing Cht on the surface of a substrate. The technique involves preparing a polymer solution using a suitable solvent; subsequently, the solution is poured onto the substrate to create a thin film deposition. This is followed by heat treatment of the substrate at a given temperature for an extended period of time to increase the adhesion strength between the substrate and the coating. Wang et al. (Wang et al., 2021) demonstrated that, through the heat-alkali treatment, an ultrathin Ti dioxide layer, upon contacting the titanium surface with air, could react with sodium hydroxide to form an amorphous sodium titanate layer, considered as the stable structure with high corrosion resistance of Ti upon contact with body fluids, precisely, a phenomenon closely related to dental implant failures.

Nevertheless, some authors have highlighted the poor cytocompatibility of Cht, which is a key factor in osteoconductivity for bone regeneration, possibly due to the lack of cell binding sites (Albrektsson and Johansson, 2001). To solve these drawbacks, the incorporation of materials such as graphene or the enrichment with bioactive materials of the extracellular matrix has been proposed (Hansson et al., 2012; Wong et al., 2020; Brun et al., 2021; Schierano et al., 2021). Saccari et al. and Fernandez et al. proposed the modification of Cht with a fibronectin-DNA complex to enhance the activity of osteoblastic cells (Fernández et al., 2012; Saccani et al., 2019).

Other studies have highlighted the degree of deacetylation and molecular weight of Cht as determinants for osteoblast adhesion, growth, and differentiation. Sukul et al. (Sukul et al., 2021), found *in vitro* that high deacetylation chitosan favored osteoblast adhesion, secretion of bone markers and extracellular matrix production, while low deacetylation induced secretion of osteoclastogenesis-promoting factors. Similarly, high molecular weight Cht induced the secretion of factors facilitating angiogenesis and bone remodeling.

These new technologies are in the research phase, and in general, although it is accepted that Cht-coated dental implants may have a higher osseointegration capacity, they are not commercially available, but are likely to become a commercial option in the future. (Bumgardner et al., 2007; Civantos et al., 2017; Palla-Rubio et al., 2019; Alnufaiy et al., 2020; López-Valverde et al., 2021b; Eftekhar Ashtiani et al., 2021; Lin et al., 2021).

## Trends; next generation coatings

Over the past 2 decades, several surface coatings approaches have been proposed and studied to improve implant osseointegration. The development of future generations of dental implants, both under normal and tissue compromised conditions, will involve “tailored modifications” of substrate surfaces that enhance their biological performance and locally release bone regeneration stimulating molecules, especially at the bone-implant interface.

The most important problems related to insufficient compatibility of material surfaces could be summarized in four types:

- Topographical or mechanical incompatibility. The implant microdesign or surface condition has a great impact on the dynamics of osseointegration, especially, in low-density bone (Steigenga et al., 2003). Although surgical integration depends mainly on the implant macrodesign, the microdesign or microsurface topography may also play an important role in this phase. Salabi et al. in an animal model demonstrated that rough surfaces presented higher removal torque values than machined surfaces (Shalabi et al., 2006). A consensus report published in 2009 concluded that “the highest level of bone-to-implant contact was associated with moderately rough surfaces” (Lang et al., 2009). Regarding macrodesign, different implant thread designs have been proposed with the aim of improving and optimizing the osseointegration process, and implant geometry has been reported to affect bone-to-implant ratio and mechanical pull-out test values (Abuhussein et al., 2010).
- Biofouling. Any artificial material introduced into the body environment is at risk of biofouling, causing infections and leading to implant failure. Numerous studies have investigated non-biofouling with the aim of increasing the bioactivity of titanium for its broader applications in biomedical areas; Kang et al. (Kang et al., 2010) used biologically active molecules on Ti surfaces coated with polyethylene glycol methacrylate to enhance the non-biofouling property. Jesmer and Wylie and Manivasagam et al. (Jesmer and Wylie, 2020; Manivasagam et al., 2021) in recent reviews highlighted that biofouling is crucial for the optimization of biomaterials and devices interacting with complex biological environments composed of macromolecules, fluids and cells.
- The reaction of the immune system to the implant material may be the cause of a number of problems. Trindade et al. and Albrektsson et al. (Trindade et al., 2016; Trindade et al., 2018) interpreted osseointegration as a foreign body reaction to a biomaterial. Macrophages, which are the main effector cells in biological reactions to biomaterials, could contribute to some extent to the success or failure of implants (Chen et al., 2016). Macrophages have also been shown to directly contribute to bone formation during the healing process (Pajarinen et al., 2019). However, some studies have reported that macrophage infiltration triggered by implant insertion promotes bone formation around implants in the first 2 weeks, whereas macrophage depletion impairs new bone formation (Wang et al., 2020). After the implantation procedure, it seems that the bone regeneration capacity is strongly activated, which would indicate that Ti implants complement the bone healing process and that innate and acquired immune mechanisms would be required in these biological events. Inflammation is the main mechanism of innate immunity; therefore, an initial and well-controlled inflammatory immune response is essential for bone formation and osseointegration (Trindade et al., 2018). Amengual-Peñafiel et al. reported in a recent review article that immunomodulatory strategies focused on host osteoimmunology are a promising approach and that osteoimmunology will allow a better understanding and integration of the concept of osseointegration in the future (Amengual-Peñafiel et al., 2021).

### Biodegradable coatings

This type of research is focused on the true biological character of osseointegration and the innovative approaches proposed are intended to mimic the biochemical environment and nanostructural architecture of human bone. Biodegradable polymers are polymers which are decomposed in a living body but whose degradation products remain in tissues for long-term. Coatings by different molecules, such as Cht, collagen, polysaccharides, peptides and biodegradable polymers, are being thoroughly investigated for their interesting results on implant surfaces (Song et al., 2020). Natural polymers possess highly organized structures and can contain an extracellular substance, called ligand, which is of great interest for binding to cell receptors; moreover, these types of structures can guide cell growth at different stages of development and stimulate an immune response (Coletta et al., 2018). It has been suggested that bioactive ions affect small molecules involved in intra- and intercellular signaling pathways and that these osteoinductive effects could be induced by bioactive small molecules that modulate bone morphogenic protein expression (Aravamudhan et al., 2013; Han et al., 2013; Kalinichenko et al., 2019).

However, although the underlying mechanism remains unexplored, the bone healing process at the bone/implant interface is hampered by oxidative stress induced by the overproduction of reactive oxygen species. In order to endow Ti substrates with antioxidant activity to enhance bone formation, Chen et al. (Chen et al., 2017) in a study on rabbit femur constructed on Ti substrates a multilayer structure composed of Cht-catechin, gelatin and HA nanofibers, obtaining excellent antioxidant and cell healing-promoting efficacy, osteogenesis differentiation and osteogenesis-related genes expression of osteoblasts.

### Laser surface treatments

Moderately rough surfaces (1–2 µm) have been reported to have benefits in osteoblast differentiation and migration (Andrukhov et al., 2016). The application of laser treatment on Ti surfaces can create identical and constant morphologies, which provides better cell adhesion and proliferation, therefore, some researchers have proposed this type of treatment on dental implants; in addition, has demonstrated its efficacy as a pre-coating with certain ceramics (Veiko et al., 2021). It has even been proposed as an alternative technology for transforming hydrophobic implant surfaces to hydrophilic (Rupp et al., 2018). When a laser is used to modify the surface properties of titanium, surface modification occurs through melting and vaporization. Different surface patterns with enhanced biological responses can be provided, and the deposition of contaminants on the surface is minimized compared to traditional modification methods (Menci et al., 2019). A systematic review by Simões et al. (Simões et al., 2021) demonstrated the ability of the laser to modify the surface properties of titanium; however, a large variation in results was observed depending on the parameters used, such as number and speed of scans, energy density, power, pulse repetition rate, fluence and focal plane variation, suggesting the need for specific protocols for the high-power laser to optimize results.

Some commercial firms have included this type of surface treatments in dental implants and prosthetic abutments. Blazquez-Hinarejos et al. (Blázquez-Hinarejos et al., 2017) in a systematic review carried out in preclinical and clinical studies, showed that different types of surface modifications of implant abutments can provide a benefit for the attachment of connective tissue to the abutment, although they recommended further human studies to obtain more evidence of the results.

### Nanotechnology

It is well established that osteogenic cells respond better to microrough Ti surfaces compared to machined surfaces, however, research is needed to find the ideal surface topography that enhances bioactivity and osteogenesis (Yeo, 2019). Nanoengineering is emerging as a field of engineering aimed at further improving the bioactivity of dental implants. It has been demonstrated, *in vitro* and *in vivo*, that surface modification of titanium implants at the nanoscale offers enhanced bioactivity, surpassing clinical microroughness, employing various strategies such as plasma treatment, micromachining, polishing/grinding, particle blasting, chemical etching, and electrochemical anodization (Chopra et al., 2021).

The American Academy of Orthopaedic Surgeons (AAOS) indicates that infection (20.4%) was the most common etiology of failure in knee arthroplasty in the United States (Veerachamy et al., 2014), so certain studies focus their research on minimizing the immediate infection of the implant during surgery, investigating on coatings and metals with antibacterial properties, since the release of some of these coatings is prolonged during the first days after implantation, however, there is still no conclusive research on these aspects. (Kunrath et al., 2020; Kunrath and Campos, 2021).

### DNA methylation

Epigenetics is the study of molecular processes that affect the flow of genetic information between DNA sequences and gene expression patterns, such as DNA methylation (Goldberg et al., 2007). The combined use of scaffolds with small molecules, such as novel epigenetic drugs (epi-drugs), can enhance cell differentiation (Cheng et al., 2016). In this regard, some studies on implant surfaces and osteoblast differentiation have focused on differences in the osteogenic potential of surfaces as a function of gene expression levels, and some have reported that implant surface topography can alter cellular activity (Zheng et al., 2020; Ichioka et al., 2021). A study by Cho et al. concluded that the surface topography of Ti implants affects their osteogenic potential through epigenetic changes, raising the possibility of using epidrugs to enhance osteogenesis on implant surfaces (Cho et al., 2021).

## Considerations

The current approach to dental implant bioengineering involves the development of functionalized surfaces and bioactive coatings, together with strategies for *in situ* drug delivery, with the aim of reducing infection rates and improving clinical outcomes. Methods have to be developed to improve osseointegration of implants, both in normal and deficient bone conditions, especially through new macroscopic designs and surface modification.

*In vitro* methods are particularly fruitful for studying biological activity mechanistically, although their clinical application remains poor, mainly due to the absence of *in vivo* biokinetics; therefore, complete replacement of animals, at least in the near future, will not be possible (Saeidnia et al., 2015).

On the other hand, orthopedic and dental implant research will require specific animal models (Table 3), which reproduce the human condition and help to understand the complex process of osseointegration, and multiple preclinical and clinical trials will be necessary before new biomedical implant designs can enter the market, focusing, above all, on long-term functional studies.

**TABLE 3:** Suitability of models and implantation sites of *in vivo* studies.

Studies	Suitability of experimental model	Model	Implantation sites
Zhang et al. (Zhang et al., 2020)	Low suitability	Rabbit	Femur
Takanche et al. (Takanche et al., 2018)	Moderate suitability	Rat	Jaw
Bumgardner et al. (Bumgardner et al., 2007)	Low suitability	Rabbit	Tibia
Kung et al. (Kung et al., 2011)	Low suitability	Rat	Subcutaneous
López-Valverde et al. (López-Valverde et al., (2021a); López-Valverde et al., (2022))	Suitable	Dog	Jaw

Likewise, imaging assessment methods will gain more and more importance as diagnostic methods for bone quantification around implant surfaces.

## Conclusion

Titanium and its alloys are the materials of choice for the manufacture of orthopedic and dental implants because of their excellent corrosion resistance and proven biocompatibility. However, there are still limitations caused by post-surgical infections or lack of biointegration.

In this review, different relevant methods of Cht coatings on Ti substrates for dental implant applications as well as next generation coatings have been addressed.

As a natural polysaccharide, it has been demonstrated in the different studies that Cht coatings provide biocompatibility, excellent adhesion and anti-corrosion properties in different coatings. In addition, it has been shown that Cht coatings offer mechanical stability during the first weeks after implantation, good degradability and optimal osseointegration. Better mineralization, cell proliferation and bioactivity have also been demonstrated around these coatings. However, despite the extensive literature, much research is needed to improve the performance of Cht-based coatings, especially by improving the bond strength and the interface between the coating and the substrate. Likewise, new coating strategies with Cht and other similar biopolymers should be implemented to improve bioactivity and adhesion without impairing the properties of the substrates, with particular attention to reducing toxicity in coating preparations.

Nevertheless, Cht-based coatings are promising candidates for improving orthopedic and dental implants, although more research is needed to develop coatings customized to each patient’s bone circumstances by establishing surfaces with a standardized topography to ensure long-term success.
